# Composite to Clarity: Shifting From Combined to Individual Endpoints in Meta-Analyses of Cardiovascular Outcome Trials

**DOI:** 10.1016/j.jacadv.2023.100548

**Published:** 2023-08-06

**Authors:** Safi U. Khan

**Affiliations:** Department of Cardiology, Houston Methodist, DeBakey Heart and Vascular Center, Houston, Texas, USA

**Keywords:** cardiovascular outcomes, endpoints, meta-analysis

Meta-analyses are crucial tools in evidence-based medicine, synthesizing disparate study results to provide robust evidence for clinical decision-making.^1^ By combining and synthesizing data from multiple studies, meta-analysis offers increased statistical power, reduces random error, and allows for the detection of treatment effects across heterogeneous populations. Therefore, a meta-analysis must offer novel insights or additional evidence, as replicating previously reported findings of individual trials diminishes its value and purpose.

The choice of endpoints in meta-analyses is a critical factor that substantially shapes the interpretation and applicability of findings. Composite endpoints such as major adverse cardiovascular events (MACE), a composite of several fatal and nonfatal cardiovascular endpoints, or Net Adverse Cardiovascular Events (NACE), a composite of cardiovascular and bleeding endpoints, have been traditionally favored in cardiovascular research for their perceived comprehensive coverage.^2^ However, their routine employment as the primary focus of meta-analysis compromises meta-analyses' interpretive and translational value.

Composite endpoints conflate various outcomes of differing severity, weight, and clinical impact into a single metric.^2^ This creates a significant potential for misrepresenting treatment effects, as the more frequent but less severe components might disproportionately shape the overall result.^3^ This can hinder the interpretability of findings and obfuscate the translation of research evidence into clinical practice. Moreover, the lack of universally accepted definitions for composite endpoints introduces variability across studies, complicating the synthesis of results in meta-analyses.

Clinical trials frequently employ composite endpoints, mainly to enhance statistical power. However, these endpoints may obscure the distinct impacts of therapy on its components,^4^ undermining the primary goals of meta-analyses: to offer effect estimates for direct application to patient care and to boost statistical power for clinically important but underpowered outcomes. Thus, replicating pooled benefits in already statistically powered composite endpoints appears redundant when focusing on individual, underpowered endpoints could yield more pertinent and patient-centered insights.

Focusing on individual endpoints allows for the capture of patient-specific values and preferences.^5^ For example, different patients might place varying importance on the different components of MACE. Some patients might value avoiding a stroke more than preventing myocardial infarction (MI). Similarly, events like MI or stroke significantly affect a patient's quality of life and life expectancy compared to less severe components of MACE, such as hospitalization for angina or revascularization. Hence, a treatment's efficacy in preventing less severe events may be overemphasized in a composite end point, whereas patients may prioritize avoiding more severe outcomes.

Analyzing individual endpoints allows for a detailed understanding of a treatment's benefits and risks, aiding in informed decision-making. For example, a treatment may reduce the risk of MI but increase the risk of severe bleeding. A composite endpoint like NACE might obscure these effects. Moreover, individual endpoints allow the exploration of heterogeneity in treatment effects across patient subgroups. For instance, treatment may reduce MI risk among older patients or those with a prior MI, while its impact might be less notable among younger patients or those without a history of MI. Again, these subtle treatment effects can be overlooked with a composite end point. Furthermore, individual endpoints can advance our understanding of disease mechanisms and treatment effects. For example, a treatment reducing the risk of cardiac death driven by a reduction in MI suggests its effectiveness in providing cardiovascular survival benefits through a reduction in MI.^6^

All-cause or cardiovascular mortality, as the primary endpoints of meta-analysis, offer a compelling alternative to the composite end point. All-cause mortality is favored over cardiovascular mortality due to its comprehensive nature and ability to capture the overall impact of treatment on survival. It reduces bias and misclassification associated with cause-specific mortality. Its universal definition reduces the potential for cross-study variability, and its importance to patients is unequivocal. That said, the selection of endpoints should also be tailored to the potential impact of the therapy on the relevant population. This involves selecting endpoints that reflect the most prevalent and clinically meaningful events in disease and captures therapy's potential benefits and harms. A balanced assessment of benefits and harms is crucial to provide a comprehensive understanding of the therapy's implications and to ensure that meta-analyses' results can inform patient-centered care.

For example, when examining the effects of antiplatelet or anticoagulation therapy, it is essential to shift the focus from composite endpoints like MACE or NACE and instead evaluate individual ischemic endpoints, MI, stroke, and cardiovascular death, as benefit endpoints. However, it is equally crucial to consider harms, including bleeding risks and drug-specific adverse effects, to thoroughly assess the potential drawbacks and risks associated with these interventions. Similarly, for therapies targeting heart failure, benefit-focused endpoints should include outcomes such as hospitalization rates, exercise tolerance, and improvements in ejection fraction. On the other hand, renal impairment, electrolyte imbalances, and medication-specific side effects should be analyzed to evaluate the potential adverse effects associated with these therapies.

In addition, analyzing patient-reported outcomes, such as quality of life, could further enhance the patient-centeredness of research. These outcomes can offer invaluable insights into the lived experience of patients, complementing traditional clinical outcomes, and making research findings more directly relevant and applicable to patients' daily lives.

In summary, while composite endpoints have a place in cardiovascular research, their limitations underscore the need for a more nuanced approach to end point selection in meta-analyses. Focus on patient-centered individual endpoints enhances the value of meta-analyses in evidence-based medicine, creating a body of research that is not only rigorous but also relevant to those it seeks to benefit—the patients.

## Funding support and author disclosures

The author has reported that he has no relationships relevant to the contents of this paper to disclose.
